# Supplementary material for: Fragmentation and low density as major conservation challenges for the southernmost populations of the European wildcat
Source: PLoS One. 2020 Jan 28;15(1):e0227708. doi: 10.1371/journal.pone.0227708 (PMC6986748; doi:10.1371/journal.pone.0227708)

**Supplementary material for**

**Fragmentation and low density as major conservation challenges for the southernmost populations of the European wildcat**.

Jose María Gil-Sánchez, Jose Miguel Barea-Azcón, Javier Jaramillo, Javier Herrera-Sánchez, José Jiménez García-Herrera & Emilio Virgós.

S1 Fig. Output of MaxEnt: ROC curve.


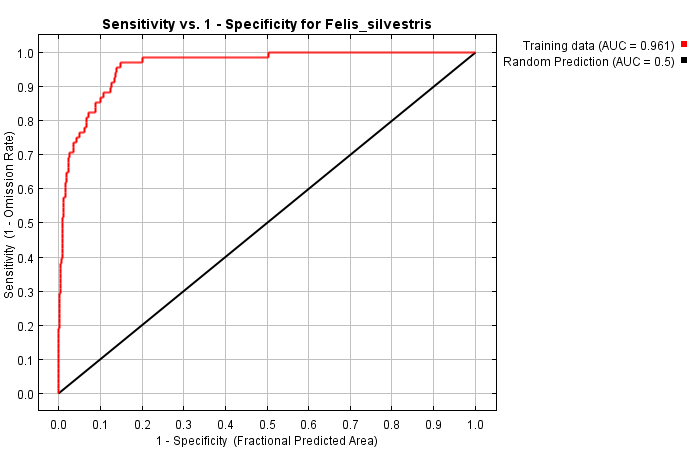

Supplement: S1 Fig — (DOCX) [file pone.0227708.s001.docx]
