# Supplementary material for: Fragmentation and low density as major conservation challenges for the southernmost populations of the European wildcat
Source: PLoS One. 2020 Jan 28;15(1):e0227708. doi: 10.1371/journal.pone.0227708 (PMC6986748; doi:10.1371/journal.pone.0227708)
Supplement: S2 Fig — (DOCX) [file pone.0227708.s002.docx]

**Supplementary material for**

**Fragmentation and low density as major conservation challenges for the southernmost populations of the European wildcat**.

Jose María Gil-Sánchez, Jose Miguel Barea-Azcón, Javier Jaramillo, Javier Herrera-Sánchez, José Jiménez García-Herrera & Emilio Virgós.

S2 Fig. Output of MaxEnt: responses of the environmental variables.

| 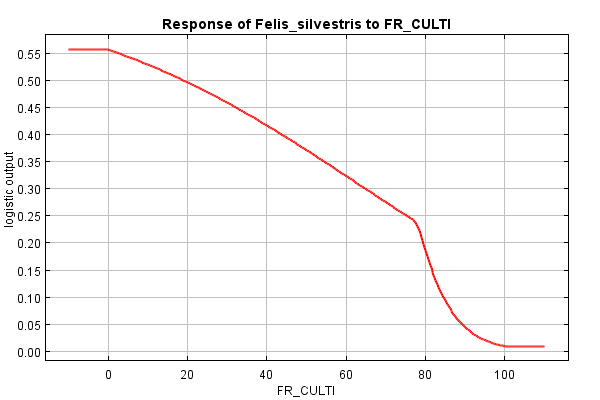 | 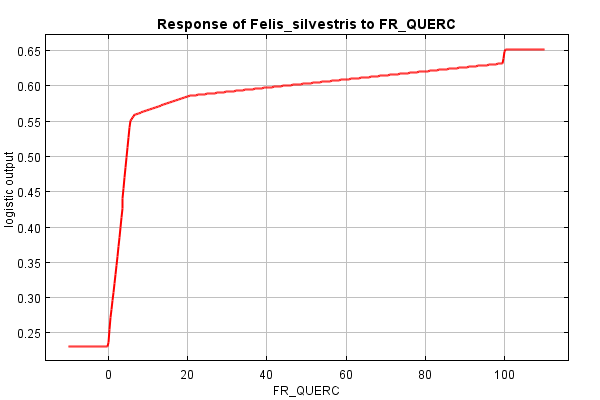 | 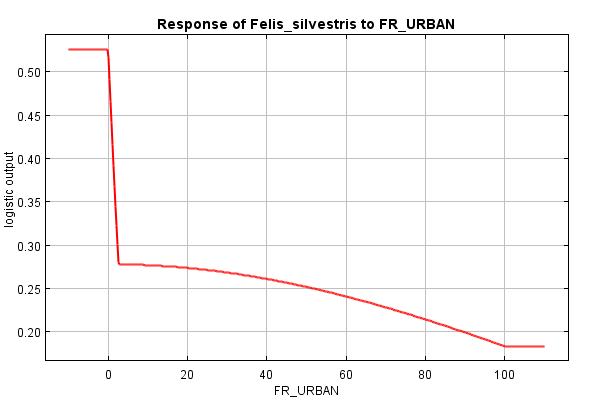 |
| --- | --- | --- |
| 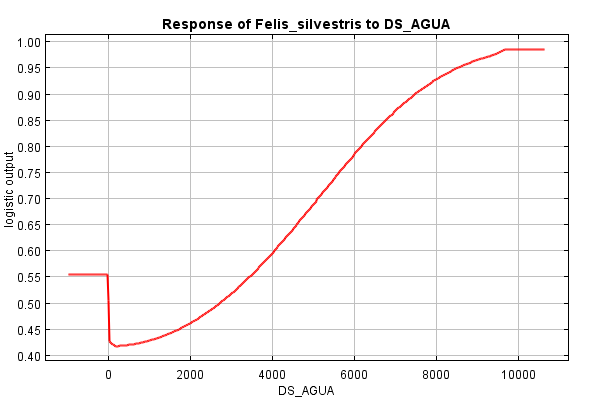 | 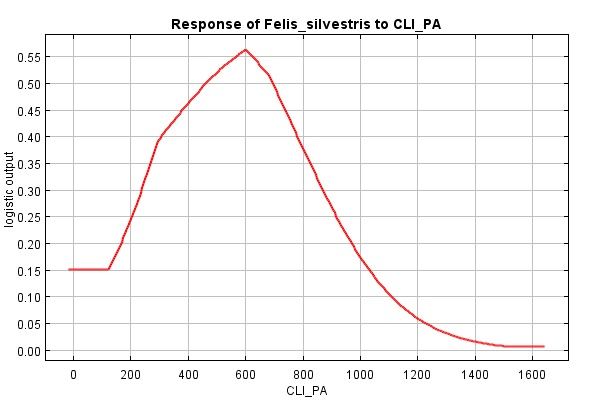 | 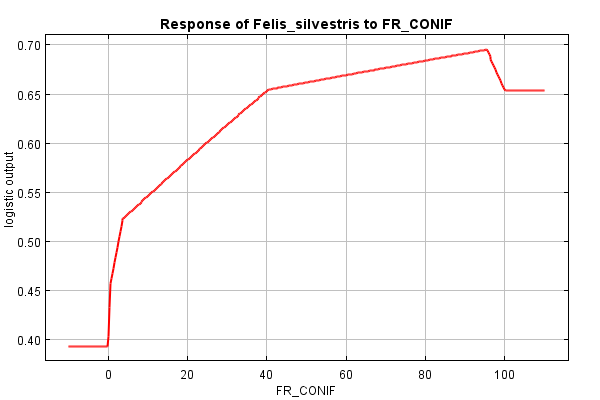 |
| 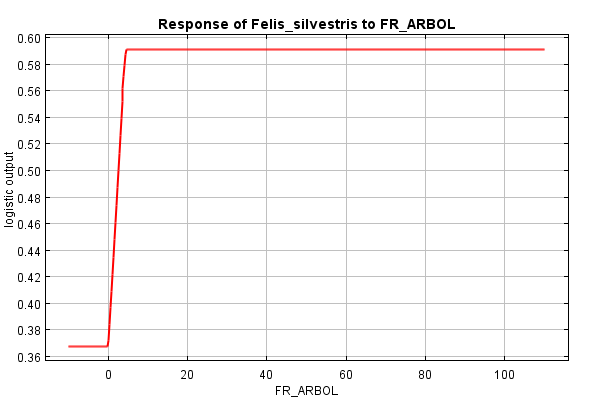 | 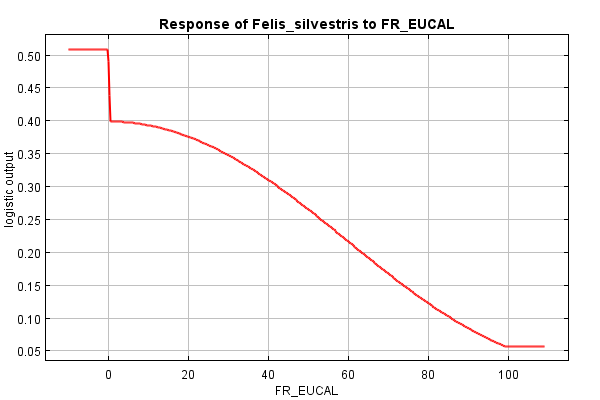 | 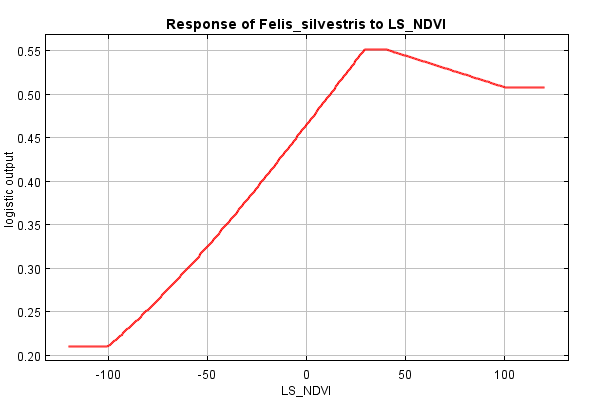 |
| 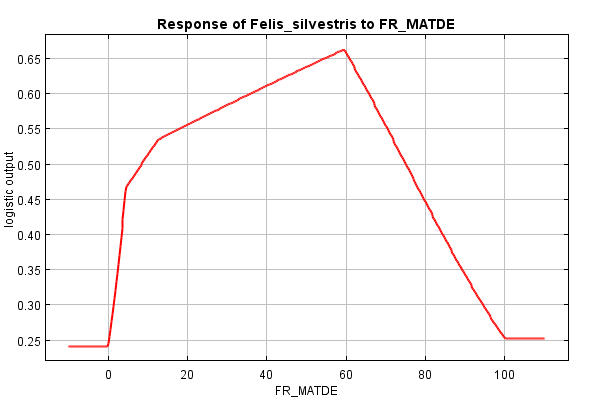 | 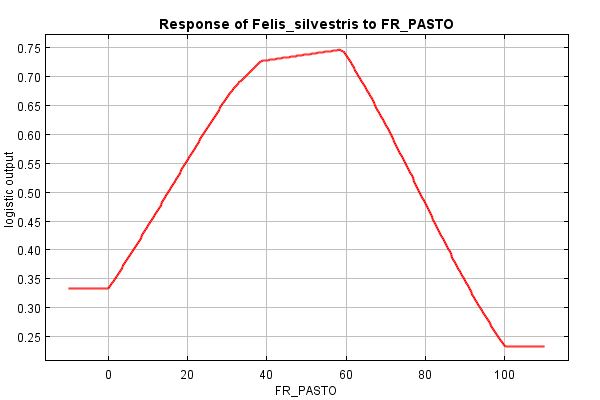 | 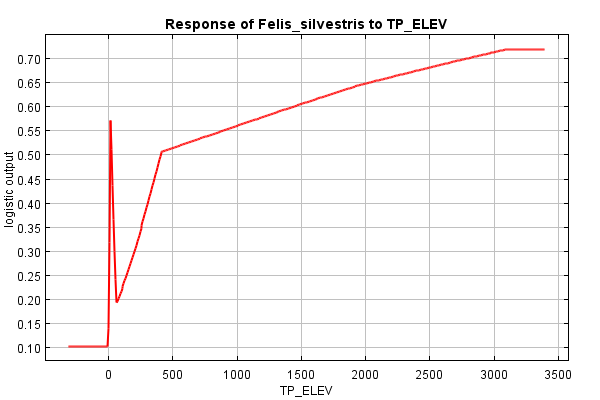 |
| 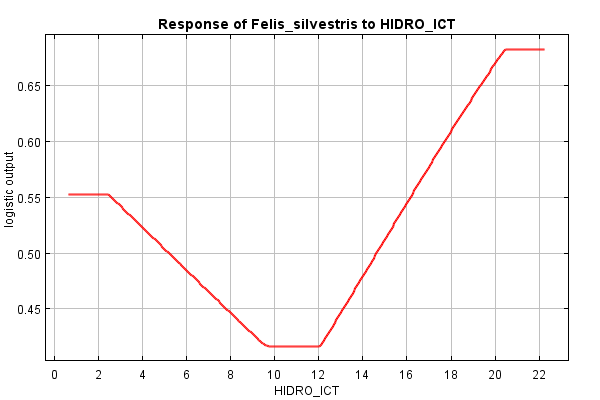 | 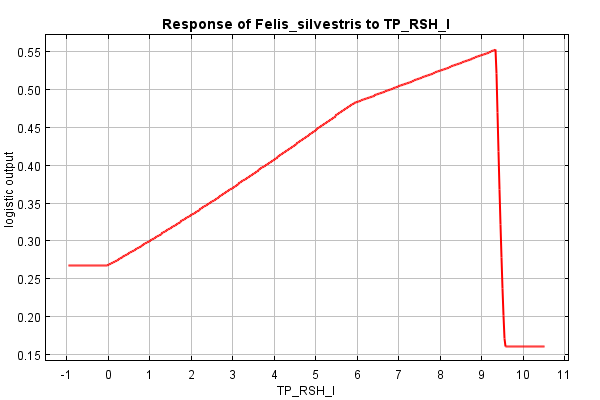 | 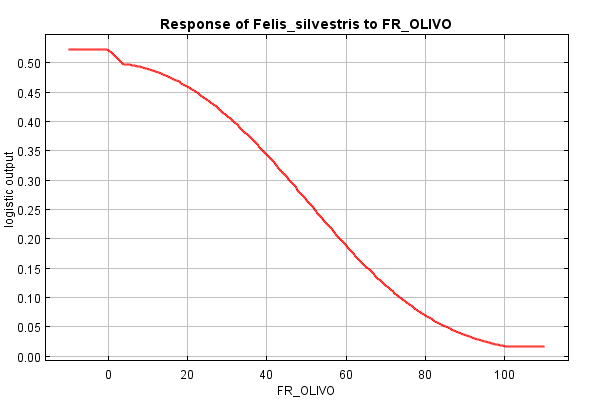 |
| 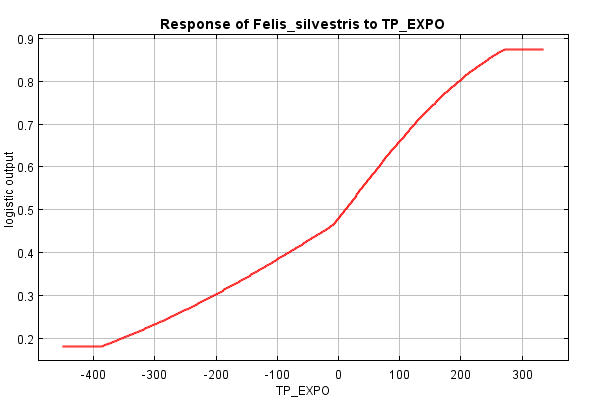 | 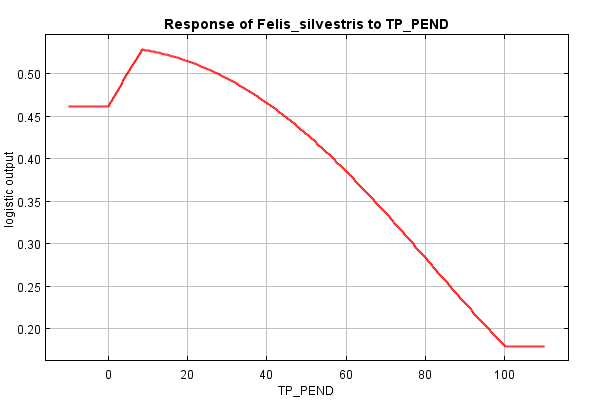 | 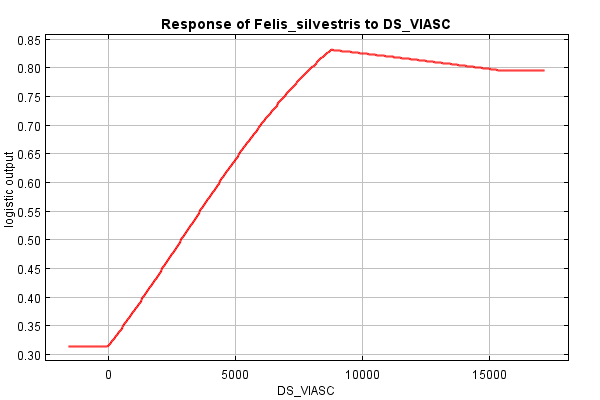 |
| 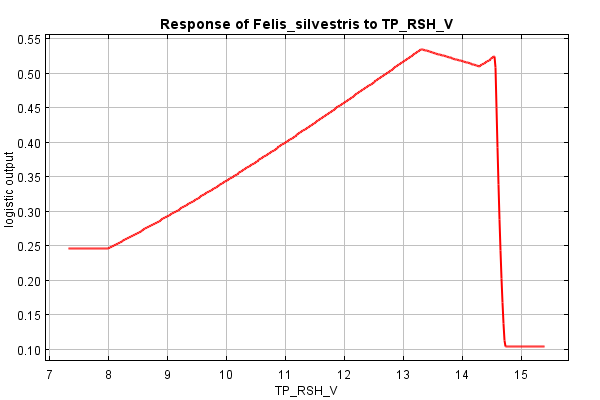 | 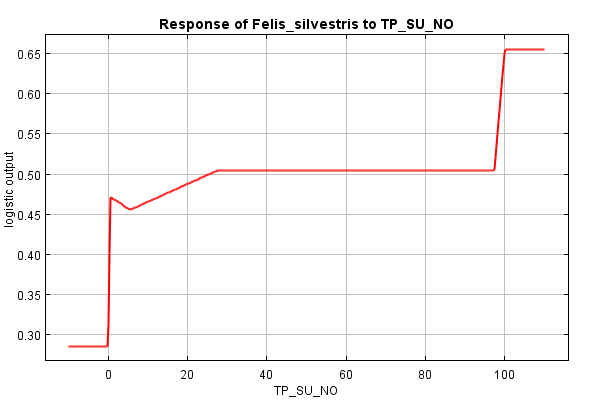 | 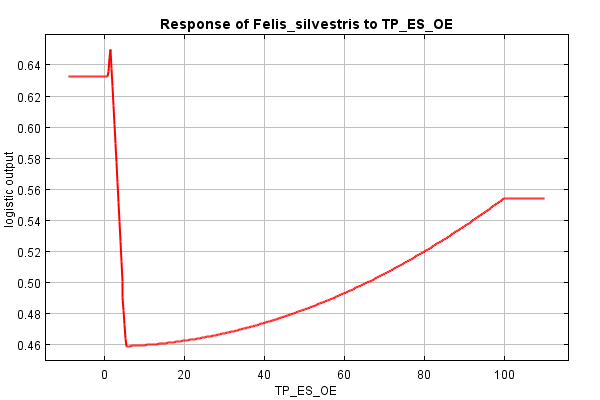 |
